# How Long and Low Can You Go? Effect of Conformation on the Risk of Thoracolumbar Intervertebral Disc Extrusion in Domestic Dogs

**DOI:** 10.1371/journal.pone.0069650

**Published:** 2013-07-24

**Authors:** Rowena M. A. Packer, Anke Hendricks, Holger A. Volk, Nadia K. Shihab, Charlotte C. Burn

**Affiliations:** 1 Department of Production and Population Health, Royal Veterinary College, University of London, Hertfordshire, United Kingdom; 2 Department of Clinical Science and Services, Royal Veterinary College, University of London, Hertfordshire, United Kingdom; 3 Southern Counties Veterinary Specialists, Unit 6, Forest Corner Farm, Ringwood, Hampshire, United Kingdom; University of Sydney, Australia

## Abstract

Intervertebral disc extrusion (IVDE) is a common neurological disorder in certain dog breeds, resulting in spinal cord compression and injury that can cause pain and neurological deficits. Most disc extrusions are reported in chondrodystrophic breeds (e.g. Dachshunds, Basset Hounds, Pekingese), where selection for ‘long and low’ morphologies is linked with intervertebral discs abnormalities that predispose dogs to IVDE. The aim of this study was to quantify the relationship between relative thoracolumbar vertebral column length and IVDE risk in diverse breeds. A 14 month cross-sectional study of dogs entering a UK small animal referral hospital for diverse disorders including IVDE was carried out. Dogs were measured on breed-defining morphometrics, including back length (BL) and height at the withers (HW). Of 700 dogs recruited from this referral population, measured and clinically examined, 79 were diagnosed with thoracolumbar IVDE following diagnostic imaging ± surgery. The BL:HW ratio was positively associated with IVDE risk, indicating that relatively longer dogs were at increased risk, e.g. the probability of IVDE was 0.30 for Miniature Dachshunds when BL:HW ratio equalled 1.1, compared to 0.68 when BL:HW ratio equalled 1.5. Additionally, both being overweight and skeletally smaller significantly increased IVDE risk. Therefore, selection for longer backs and miniaturisation should be discouraged in high-risk breeds to reduce IVDE risk. In higher risk individuals, maintaining a lean body shape is particularly important to reduce the risk of IVDE. Results are reported as probabilities to aid decision-making regarding breed standards and screening programmes reflecting the degree of risk acceptable to stakeholders.

## Introduction

### Chondrodystrophy and Intervertebral Disc Extrusion (IVDE)

Intervertebral disc extrusion (IVDE) is the most common spinal neurological disorder in domestic dogs [1]. IVDE can result in spinal cord compression and injury which may be associated with pain, sensory and motor deficits, that can significantly compromise quality of life [2]. In severe cases this may result in permanent loss of function, with owners choosing to euthanise their dog or nurse them long-term as paraplegics [3], in some cases using carts for mobility [4]. Most disc extrusions occur in chondrodystrophic breeds, with a recent large-scale epidemiological study finding that all seven of the breeds at highest risk of thoracolumbar IVDE were chondrodystrophic [5].

In chondrodystrophic dogs, altered epiphyseal chondroblastic growth and maturation results in disproportionate dwarfism [6], manifested as a ‘long and low’ morphology. In these breeds, IVDE commonly occurs between 3–7 years of age [7], following a degenerative process, chondroid metaplasia. During this process the nucleus pulposus (NP) changes from being jelly-like to hardened and calcified, with diminished shock-absorbing capabilities [8], and impaired abilities to dissipate forces acting on the vertebral column [9]. During extrusion, rupture of the dorsal annulus fibrosis (AF) leads to extrusion of this degenerated disc material into the spinal canal. This is in contrast to disc protrusion, where dorsal bulging of the AF and NP can lead to impingement of the spinal cord; however, the NP is retained within the AF. Disc protrusions were previously thought to be caused by a different pathophysiological process to chondroid metaplasia, termed fibroid metaplasia [10]; however, more recent studies have questioned this distinction, and found similarities between these processes [11], [12], [13]. Despite this, the demographics of dogs affected by these two types of disc disease are divergent, with differences in breed and age, and also characteristics of the diseases such as speed of disc degeneration, frequency of disc calcification and spinal level affected, as recently reviewed [14].

### Chondrodystrophy in Breed Standards

The development of chondrodystrophic breeds of dog has been by direct selection for the pathological trait of disproportionate dwarfism [1], with concomitant but unintentional selection for the associated intervertebral disc abnormalities predisposing them to IVDE. The strong phenotypic relationship between chondroid metaplasia and this form of disproportionate dwarfism, and the abnormal chondrocyte differentiation observed in both settings, suggest that chondroid metaplasia is largely attributable to a pleiotropic effect of the hypochondrodysplasia gene [15].

At least 26 breeds have been referred to as chondrodystrophic in the recent veterinary literature (full results of a systematic search between 2007–2012 documented in Table 1). Classification is often inconsistent, as a systematic investigation of the intervertebral discs of many short-legged breeds has not been carried out, to ascertain whether they show characteristic histological abnormalities. It has been suggested that chondrodystrophic dogs can be distinguished from non-chondrodystrophic breeds based on their ‘long and low’ physical appearance [11]. Chondrodystrophy is therefore implicitly written into the breed standards of several breeds by describing the long and low morphology; e.g. the Dachshund breed morphologies are described using the terms “*forearm short*”, “*lower thigh short*” in comparison to body “*moderately long*” (with ‘moderately’ added in 2009 following breed standard revisions to discourage exaggeration) [16]. Similarly, the Basset Hound’s body is described as “*long and deep throughout length*” and “*forelegs short*” [17].

**Table 1:** Breeds referred to as chondrodystrophic in recent publications.

Breed	Number of papers	References
Standard Smooth Haired Dachshund	20	[11], [44], [45], [46], [47], [48], [49], [50], [51], [52], [53], [54], [55], [56], [57], [58], [59], [60], [61], [62]
Standard Long Haired Dachshund	20	[11], [44], [45], [46], [47], [48], [49], [50], [51], [52], [53], [54], [55], [56], [57], [58], [59], [60], [61], [62]
Miniature Wire Haired Dachshund	20	[11], [44], [46], [47], [48], [49], [50], [51], [52], [53], [54], [55], [56], [57], [58], [59], [60], [61], [62], [63]
Miniature Smooth Haired Dachshund	19	[11], [44], [46], [47], [48], [49], [50], [51], [52], [53], [54], [55], [56], [57], [58], [59], [60], [61]
Miniature Long Haired Dachshund	19	[11], [44], [46], [47], [48], [49], [50], [51], [52], [53], [54], [55], [56], [57], [58], [59], [60], [61], [62]
Standard Wire Haired Dachshund	20	[11], [45], [46], [47], [48], [49], [50], [51], [52], [53], [54], [55], [56], [57], [58], [59], [60], [61], [62]
Beagle	11	[11], [46], [47], [49], [50], [54], [56], [57], [58], [60], [64]
Shih Tzu	7	[11], [44], [46], [47], [55], [59], [61]
Cocker Spaniel	7	[44], [46], [47], [52], [58], [59], [65]
French Bulldog	6	[11], [48], [50], [52], [61], [62]
Pekingese	6	[44], [46], [49], [50], [58], [62]
Miniature Poodle	5	[11], [46], [47], [48], [56]
Basset Hound	4	[11], [44], [49], [63]
Corgi (Unspecified)	3	[11], [44], [49]
Lhasa Apso	3	[44], [46], [55]
Jack Russell Terrier	2	[11], [59]
Yorkshire Terrier	2	[48], [59]
Pug	1	[11]
English Bulldog	1	[11]
Bichon Frise	1	[46]
Maltese	1	[46]
Coton de Tulear	1	[52]
Havanese	1	[55]
West Highland White Terrier	1	[66]
Sealyham Terrier	1	[62]
Boston Terrier	1	[62]

To gain a consensus view on which dogs were classed as chondrodystrophic, Web of Science was systematically searched using the terms “((dog* OR canine) AND chondrodystroph*)”, with full scientific papers published in English between 2007–2012 included. The full text of all 24 papers returned by the search were examined to determine which breeds were explicitly referred to as chondrodystrophic.

### Risk Factors for Disc Calcification and Extrusion

Chondrodystrophy has recently been found to be associated with the expression of a retrogene encoding fibroblast growth factor 4 (FGF4) located on chromosome 18 [18]. If being chondrodystrophic confers an increased risk of IVDE, then it may be expected that all dogs exhibiting this trait are at an equal risk of developing this disorder. However, a continuous spectrum of disc degeneration and extrusion is seen both among and within breeds, suggesting a multi-factorial aetiology involving cumulative effects of several genes (including those selected for the chondrodystrophic conformation), and environmental factors. In Dachshund-specific studies, environmental factors implicated as either increasing or decreasing the risk of disc disease [19] include biomechanical factors related to the dog and its physical form, the environment it lives in, and the lifestyle it leads. Careful interpretation of results is required here, as risk factors must be considered in relation to whether they increase the risk of disc calcification in isolation (potentially preceding an extrusion event) or the actual disc extrusion event, as biomechanics may differentially affect these processes.

Disc calcification rates are reduced by increased duration of exercise and moderate, rather than infrequent, stair climbing [20]. However, in that study most activity-related environmental factors had no significant influence upon disc calcification, which may reflect the relatively high contribution of genetics to the chondrocyte abnormalities in the intervertebral disc. The study also investigated the influence of conformation, including relative back length, on disc calcification, but no effect of bodily dimensions could be found. The authors however conceded that this non-significance may be due to other (likely genetic) factors being of a more major significance than morphological factors to calcification, with their moderately small sample size (n = 48) meaning more subtle effects were less likely to be demonstrated [20]. The study was additionally limited to a Standard Wirehaired Dachshund population, which is likely to exhibit less variability than a study involving a variety of chondrodystrophic and non-chondrodystrophic breeds, where differences in risk may be detected across the phenotypic spectrum.

Disc extrusions, on the other hand, are often induced by normal day-to-day canine activities, such as jumping and climbing stairs [9]. Extrusions are observed most frequently at high-motion sites in the vertebral column, such as the thoracolumbar junction [1], [10]. As stated by Verheijen and Bouw (1982), there is a liability to larger bending moments in a long back if the bending is not spread over the entire vertebral column in an even fashion leading to additional ‘wear and tear’ on certain discs during bending, flexion and torsion [21]. If increased back length relative to leg length is a risk factor additional to having chondrodystrophic genetics, then that could explain the high prevalence and overrepresentation of IVDE in Dachshunds, a group of breeds exhibiting some of the most extreme back length to leg length ratios, with a relative risk of IVDE 10–12 times higher than other breeds [7], [22], and 19–24% of Dachshunds estimated to be affected during their lifetime [19], [22]. A recent breed club led survey of UK Dachshunds reported an average intervertebral disc disease prevalence of 6.8% across all varieties; however, it was 15.3% in the Standard Smooth Haired Dachshund [23]. Dachshunds are the breeds most likely to develop a recurrence of clinical signs after surgery [24], [25], [26], [27], [28]. In Dachshunds, relatively longer dogs experience the most severe clinical signs when affected by thoracolumbar IVDE [28].

Despite this, when physical characteristics (including conformation, body weight and body condition score) of Dachshunds with clinically confirmed or suspected disc extrusions and disc protrusions were compared to those unaffected by this disease, no effect of relative back length was found [28]. However, that study included dogs with both disc extrusions and protrusions, and it is possible that morphology has a differing role in these events. Furthermore, the study was limited to a moderately small population of Dachshunds with (n = 39) or without (n = 36) confirmed or presumed IVDE, so does not provide information of the risk of disc extrusions across the entire conformational spectrum of relative back lengths, independent of breed.

The aim of the current study was therefore to investigate the relationship between relative thoracolumbar vertebral column (‘back’) length and intervertebral disc extrusion (IVDE) in a wide range of morphologically diverse breeds focussing on IVDE in the thoracolumbar vertebral column only, which is more likely to be directly affected by back and leg morphology than the cervical vertebral column, and is most commonly affected, with 85% of cases found in this region [29]. If relatively longer backs and other aspects of morphology confer an increased risk of thoracolumbar IVDE, risk estimates for different morphologies will be generated from this hospital population, to help develop limits within breed standards to avoid IVDE and improve canine welfare, as has been previously suggested by the Council of Europe [30].

## Materials and Methods

### Recruitment of Owners and Study Dogs

Between December 2010 and January 2012, every dog referred to the Royal Veterinary College Small Animal Referral Hospital (RVC SARH) was considered for inclusion in the study. Owners of dogs referred to any clinical service for a routine appointment were approached. As appointments were booked in advance, all dogs were considered for recruitment prior to their arrival at the hospital and were excluded on a case-by-case basis if they were:

- Presented for a disorder that would make them unsuited to leaving wards/nursing care during their stay in the hospital, or too painful/uncomfortable to be handled. (N.B. New IVDE cases, that often present with an acutely painful spine, were recruited but not handled until 3–5 days post-surgery when receiving analgesia)
- Known to be aggressive and therefore not suitable for handling
- Isolated from the general hospital population for infection control
- Already recruited to a separate clinical trial/study within the study hospital

The owners of the remaining dogs (n = 700) were approached in the waiting room before their consultation, to request consent.

### Ethics Statement

This study was approved by the RVC’s Ethics and Welfare Committee (reference number: URN 2010 1054).

### Morphology

Morphometric data were collected for each dog using previously defined protocols [31], measuring 11 conformational features that were demonstrated to be breed-defining: muzzle length, cranial length, head width, eye width, neck length, neck girth, chest girth, chest width, body length (from sternum to rear of thigh), height at the withers (HW) and height at the base of tail (all in cm). All measures were taken to the nearest millimetre. HW was measured from the midpoint of the withers to the floor with a stadiometer in the standing dog. For this study, an additional measure, ‘back length’ (cm) (thoracolumbar vertebral column length), was included. Back length (BL) was measured from the mid-point of withers to the sacrum to encompass the thoracolumbar vertebral column in the standing dog. The mid-point of the withers was identified by palpation of the proximal borders of the scapulae, and the sacrum was identified by palpation of the lumbosacral space, between the dorsal processes of L7-S1. The distance between the withers and the sacrum was measured using a hard 1m ruler to measure the linear distance between these two landmarks. The BL:HW ratio was then calculated for each dog by dividing BL by HW. Weight (kg) was measured in all dogs on regularly calibrated digital scales, and body condition score (BCS) was assessed on the Purina 9 point scale [32] by a single-rater (RMAP).

Although the BL:HW ratio was the morphological predictor of interest, other aspects of morphology may have an influence on IVDE risk; so other bodily dimensions were taken into account. Principal Component Analysis of the remaining measurements was carried out, to attempt to replicate two variables previously identified from a similar canine morphometric data set [31]. These variables explained overall skeletal body size (PC1) and ‘thickness’ or ‘broadness’ of the dog (PC2). Back length (measured instead of ‘body length’ as defined by Sutter *et al*. (2008) and height at the withers were omitted from this analysis, so these variables were not included in the statistical models twice. Principal components were extracted based on eigenvalue, with only those greater than one extracted. As such, only one principal component was extracted here (PC1, eigenvalue of 8.05), explaining 73.2% of variance in the 11 variables, and all component loadings were positive.

### Clinical Classification

Clinical data for each dog recruited to the study were extracted from the RVC Clinical Record Information System, to determine whether dogs were affected or unaffected by this disease. Dogs were classed as ‘affected’ by IVDE by either changes consistent with extruded disc material on diagnostic imaging (magnetic resonance imaging, myelography, computed tomography or computed tomography myelography) and/or surgically confirmed extruded disc material in the canal of the vertebral column. Dogs suspected to have a disc lesion following neurological examination, but without further imaging or surgery were classed as ‘suspected’. Due to the uncertainty of their diagnostic status, suspected cases were excluded from further analyses to increase confidence in the results. The remaining dogs that on examination were not suspected to be clinically affected by IVDE (absence of spinal hyperesthesia and/or clinical signs of a myelopathy), and with no history of this disease or spinal hyperesthesia were classed as ‘unaffected’. As not all dogs in the hospital population underwent advanced diagnostic imaging of the vertebral column; whether disc degeneration was present in unaffected dogs, without clinical signs of disease is unknown. Dogs affected by intervertebral disc disease were assigned to categories based on the location of the affected disc(s) (cervical, thoracolumbar or lumbosacral) and on the type of disc disease detected (extrusion or protrusion).

### Statistical Analysis

Data were analysed using generalised linear mixed models for binary outcomes in R, using lmer from the lme4 package. Being affected by a thoracolumbar IVDE (binary) was the response variable, with dogs affected by other disc disease types (protrusions and cervical) excluded from the analysis. BL:HW ratio, age, PC1 and BCS were modelled as continuous fixed effects. Breed was included as a random effect, with the top 40 most popular breeds in the study population coded individually using Kennel Club nomenclature. The dogs of the remaining breeds were coded into groups using their Kennel Club breed groupings, e.g. Other Toy, Other Utility etc. All Miniature Dachshund varieties (long, smooth and wire haired) were combined as ‘Miniature Dachshund’. All cross breeds were coded plainly as ‘cross breed’ due to the unknown parentage of many of these dogs. This random effect took into account the genetic non-independence of multiple members of the same breed in the study population, and possible demographic and environmental factors, such as owners of some breeds being more likely to live in certain areas (e.g. rural *vs*. urban), or housing types (e.g. apartments vs. houses). Non-morphometric predictors i.e. signalment: age, sex, neuter status and Parker genetic/Kennel Club grouping [33], [34] were tested in all models.

Multicollinearity was checked for in all models, identified from inflated standard errors in the models, and thus avoided. Model fit was assessed using the deviance and Akaike's information criterion. From the model output, equations were used to calculate the probability of being affected by IVDE at different values of BL:HW ratio and BCS, using breed-specific random effects to compare different breeds’ risks. For the variables held constant in the model whilst the fixed effect under investigation was varied, the mean value was used for that breed, to represent an average member of the breed.

To compare high risk breeds to generic cross breed dogs, quartiles of PC1 were calculated, and the 25^th^, 50^th^ and 75^th^ percentiles were used as values for PC1 for examples of small (−0.86), medium (0.12) and large (0.82) cross breeds, respectively. Breeds with PC1 values close to these values include the West Highland White Terrier (breed mean −0.82) as a small breed, Border Collie (breed mean 0.13) as a medium breed, and Dalmatian (breed mean 0.83) as a large breed. To compare high risk breeds to generic cross breed dogs of differing back lengths, the BL:HW ratio spectrum of this population (0.54–1.83) was equally split into four groups, with cut-offs at 0.86, 1.19 and 1.51 to represent relatively short, relatively long, and extreme relatively long morphologies.

## Results

### Overall Population Demographics

A total of 700 dogs were included in the study population between December 2010 and January 2012. Of these, 13% were cross breeds and 87% pure bred. Ninety-seven breeds were represented, with the five most common being the Labrador Retriever (56 dogs, 8%), German Shepherd Dog (36 dogs, 5.1%), Dachshund, Miniature Smooth Haired (32 dogs, 4.6%), Pug (32 dogs, 4.6%) and Border Collie (28 dogs, 4%). Exactly three hundred dogs were female (43%) versus 400 males (57%) with the majority neutered (505 dogs, 72%). The mean±SE weight (kg) was 21.5±0.55, with the median BCS 5 (range: 2–8). Almost half (46%) of dogs were overweight (BCS>5). The mean±SE age was 5.17±0.13 years (range 3 months –15 years 3 months).

### Affected Population Demographics

There were 129 dogs diagnosed with, or suspected to have some form of disc disease, with 79 confirmed to have a thoracolumbar IVDE following detection of extruded disc material in the spinal canal via diagnostic imaging ± surgery. The most common site of IVDE was between T13-L1 vertebrae (the thoracolumbar junction) with 21% of all confirmed IVDE occurring in this location, followed by 17% at T12–13. Of the dogs affected by thoracolumbar IVDE, 84% were pure bred (representing 22 breeds) and 16% were cross breeds (Table 2). The breed most commonly presented was the Miniature Smooth Haired Dachshund (27% of cases), followed by cross breeds (16%), Cocker Spaniels (9%), Jack Russell Terriers (8%) and Miniature Long Haired Dachshunds (6%) (Table 3). The four represented Dachshund breeds (the two aforementioned plus Miniature Wire Haired and Standard Smooth Haired) comprised 38% of cases combined (30/79 cases). Affected cross breeds included chondrodystrophic crosses such as the ‘Basschund’ (Basset×Dachshund), ‘Jackshund’ (Jack Russell×Dachshund), and ‘Puggle’ (Pug×Beagle). Other demographics are reported in Table 3.

**Table 2:** Comparison of signalment between dogs affected and unaffected by thoracolumbar intervertebral disc extrusions.

	Classification	
Category	Unaffected	Affected
Total number	571	79
Pure Bred (%)	87.0	83.5
Female (%)	45.0	40.5
Neutered (%)	71.0	77.0
Median BCS, Range	5 (2–8)	5.5 (4.5–7.5)
Age (years)mean ± SE (95% CI)	4.89±0.14 (4.6–5.16)	6.14±0.34 (5.45–6.82)
Weight (kg)mean ± SE (95% CI)	23.3±0.62 (22.1–22.5)	12.3±0.98 (10.3–14.2)

The mean BL:HW ratio was higher in the affected group (1.28, 95% CI: 1.22–1.34) *vs.* the unaffected group (1.03, 1.02–1.05). When considering BL:HW ratios by breed, the most extreme breed was the Miniature Long Haired Dachshund with a mean±SE of 1.66±0.03, in contrast to the lowest mean BL:HW in the Standard Poodle, with a mean± SE of 0.71±0.17 (Table 3). Ten of the 15 longest breeds were affected by IVDE; however, the remaining ‘unaffected’ 5 breeds were represented by six dogs or less per breed, so in a larger population may also have been affected. A further 12 breeds, and cross breeds were also affected despite being of more moderate BL:HW ratios.

**Table 3:** Mean BL:HW ratios of the 15 longest breeds, and other breeds affected by thoracolumbar IVDE, with number diagnosed with IVDE.

	Breed	Mean BL:HW ratio	SE	N	N (%) diagnosed with IVDE
**The 15 longest breeds**	Dachshund, Miniature Long Haired	1.66	0.03	16	5 (32%)
	Dachshund, Standard Long Haired	1.64	–	1	0 (0%)
	Dachshund, Standard Smooth Haired	1.59	0.01	2	1 (50%)
	Dandie Dinmont Terrier	1.59	–	1	1 (100%)
	Pekingese	1.57	0.04	3	2 (67%)
	Pembroke Welsh Corgi	1.52	0.26	2	0 (0%)
	Dachshund, Miniature Smooth Haired	1.51	0.03	32	21 (65%)
	Basset Hound	1.40	0.03	7	1 (14%)
	Dachshund, Miniature Wire Haired	1.38	0.04	3	3 (100%)
	Coton de Tulear	1.34	–	1	1 (100%)
	Cardigan Welsh Corgi	1.31	0.03	2	1 (50%)
	Shih Tzu	1.30	0.03	13	3 (23%)
	Lhasa Apso	1.29	0.09	4	0 (0%)
	Bichon Frise	1.28	0.05	6	0 (0%)
	Chinese Crested	1.25	–	1	0 (0%)
**Other affected breeds**	Shetland Sheepdog	1.18	–	1	1 (100%)
	Cavalier King Charles Spaniel	1.10	0.02	26	2 (8%)
	Clumber Spaniel	1.09	–	1	1 (100%)
	Cocker Spaniel	1.04	0.02	18	7 (39%)
	Bulldog	1.04	0.04	16	1 (6%)
	German Shepherd Dog	1.04	0.07	36	2 (6%)
	Labrador Retriever	1.03	0.01	57	1 (2%)
	Cross Breed	1.02	0.02	93	13 (14%)
	Border Collie	1.01	0.02	28	3 (11%)
	Jack Russell Terrier	0.99	0.02	21	6 (29%)
	Rottweiler	0.99	0.04	12	1 (8%)
	Staffordshire Bull Terrier	0.95	0.03	16	1 (6%)
	Pomeranian	0.92	0.03	6	1 (17%)

### Risk Factors for IVDE

Three continuous variables were significantly associated with the risk of thoracolumbar IVDE; BL:HW ratio, PC1 and BCS (Table 4). Both BL:HW ratio and BCS were positively associated with risk of IVDE, indicating relatively longer and increasingly overweight dogs were at an increased risk of IVDE. In contrast, an increase in PC1 resulted in a decreased risk of IVDE, indicating skeletally smaller dogs were at higher risk. With each year increase in age there was an increased risk of being affected of IVDE; however, this was not significant (*p* = 0.053) but the inclusion of age improved model fit. No effects of sex, neuter status, or Kennel Club/Parker genetic grouping were found in any model, and were not found to improve model fit (determined by AIC values and % correct classification), and as such they were not included in the final model. When examining random effects coefficients for each breed, those with the highest additional breed-specific effects were the Miniature Dachshunds, Jack Russell Terrier and Cocker Spaniel.

**Table 4:** Results of binary response mixed model analysis of key predictors upon the risk of thoracolumbar intervertebral disc extrusion.

Predictor	Odds Ratio (95% CI OR)	SE (coef)	z	*p*
Back length : height at the withers ratio (BL:HW)	50.3 (7.58–333.9)	0.96	4.06	<0.001
PC1	0.56 (0.36–0.87)	0.23	−2.60	0.009
BCS	1.62 (1.14–2.31)	0.18	2.68	0.007
Age	1.10 (0.99–1.20)	0.05	1.93	0.053

**Figure 1:**
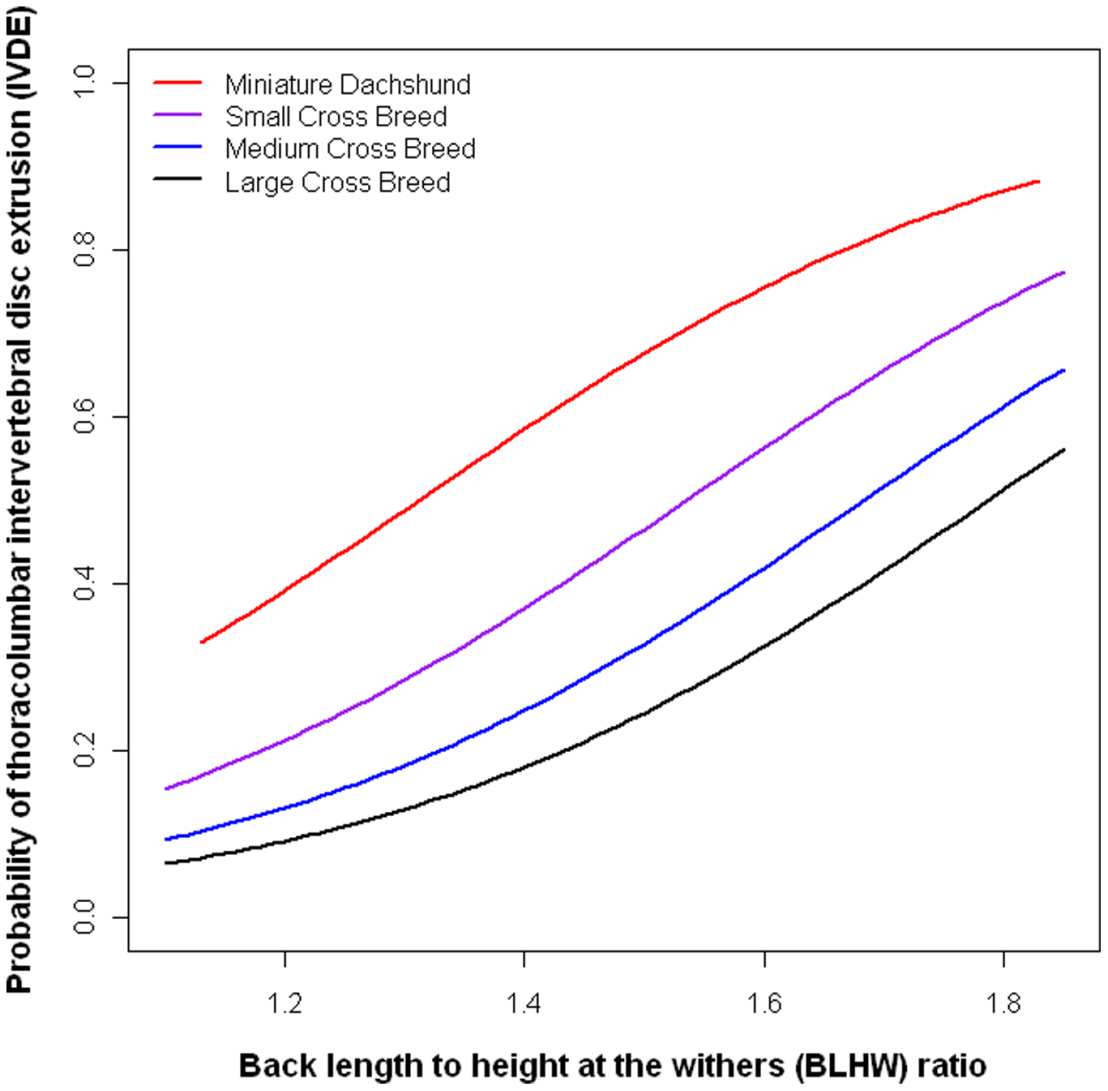
Probability of being affected by thoracolumbar intervertebral disc extrusion across the relative back length spectrum. The risk of thoracolumbar intervertebral disc extrusion is demonstrated here across the back length to height at the withers (BL:HW) ratio scale. This is demonstrated in the Miniature Dachshund breed (red) and small (purple), medium (blue) and large (black) cross breeds. As BL:HW increases, probability of being affected increases in all breeds. Age = 5 years and BCS = 5 for all dogs.

The mean BL:HW ratio of the Miniature Dachshund breeds was 1.5, with the highest recorded BL:HW in the study also that of a Miniature Dachshund, at 1.83. The risk of IVDE was highest in Miniature Dachshunds, and because our study population contained a reasonably large sample of these breeds, breed-specific risks have been calculated here (they have not been calculated for other breeds exhibiting this morphology because the sample sizes were small (see Table 3)). The effect of increased BL:HW ratio is demonstrated in Figure 1, for Miniature Dachshunds, and generalised small, medium and large cross breeds.

**Figure 2:**
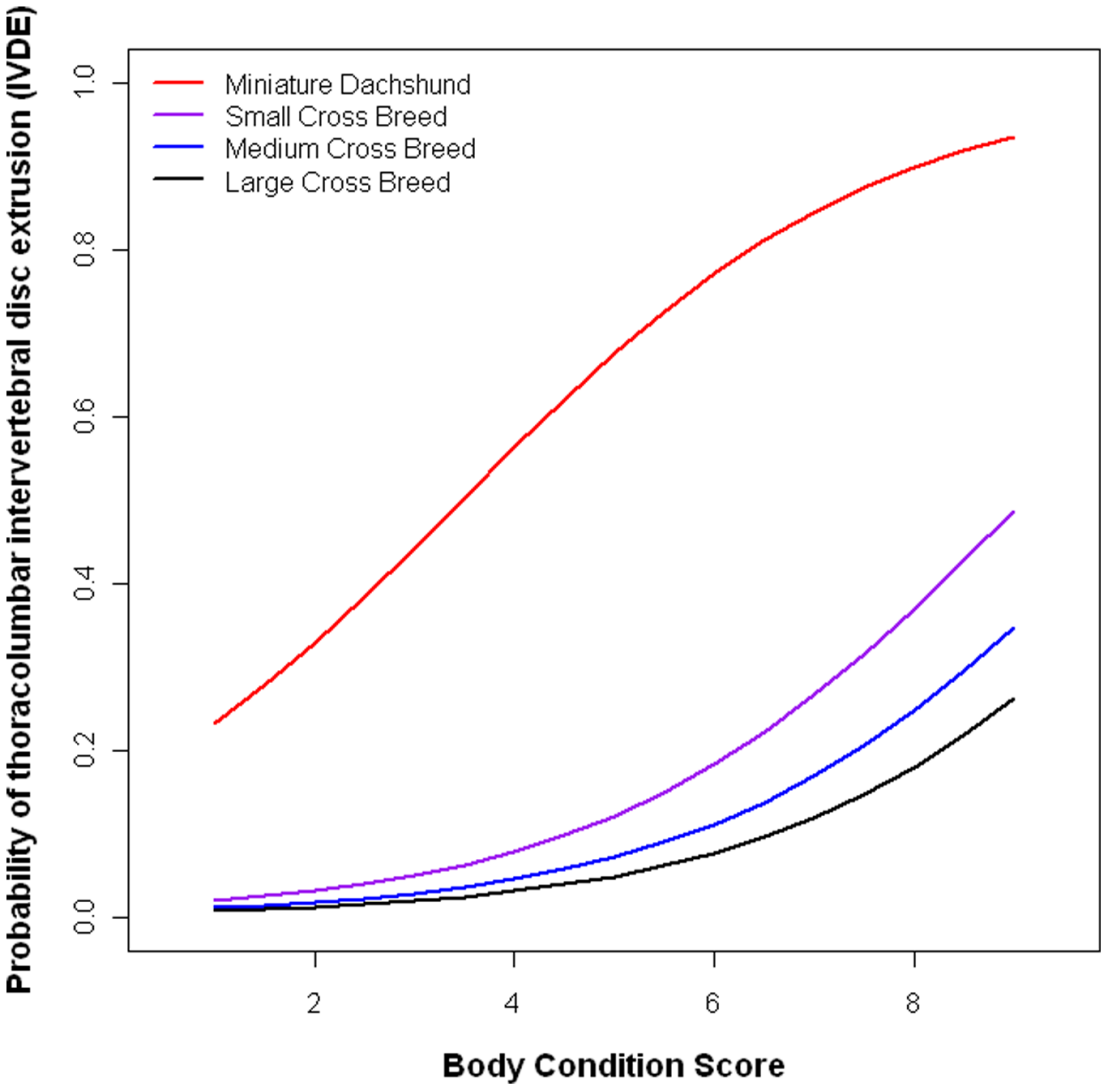
Probability of being affected by thoracolumbar intervertebral disc extrusion across the body condition score scale. The risk of thoracolumbar intervertebral disc extrusion is demonstrated here across the body condition score (BCS) scale, using the Purina 9 point system. This is demonstrated in the Miniature Dachshund breeds (red), and small (purple), medium (blue) and large (black) cross breeds. As BCS increases, probability of being affected increases in all breeds. Age = 5 years and BL:HW = 1.5 (breed mean) for Miniature Dachshunds, and age = 5 years and BL:HW = 1.03 (breed mean) for all cross breeds.

IVDE risk is increased in obese dogs, but in high-risk breeds (as exemplified here by the Miniature Dachshund), even being moderately overweight increases the risk (Figure 2). Nearly half (46.2%) of Miniature Dachshunds in the study population were overweight (BCS>5), with 13% substantially overweight at BCS 7.

**Figure 3:**
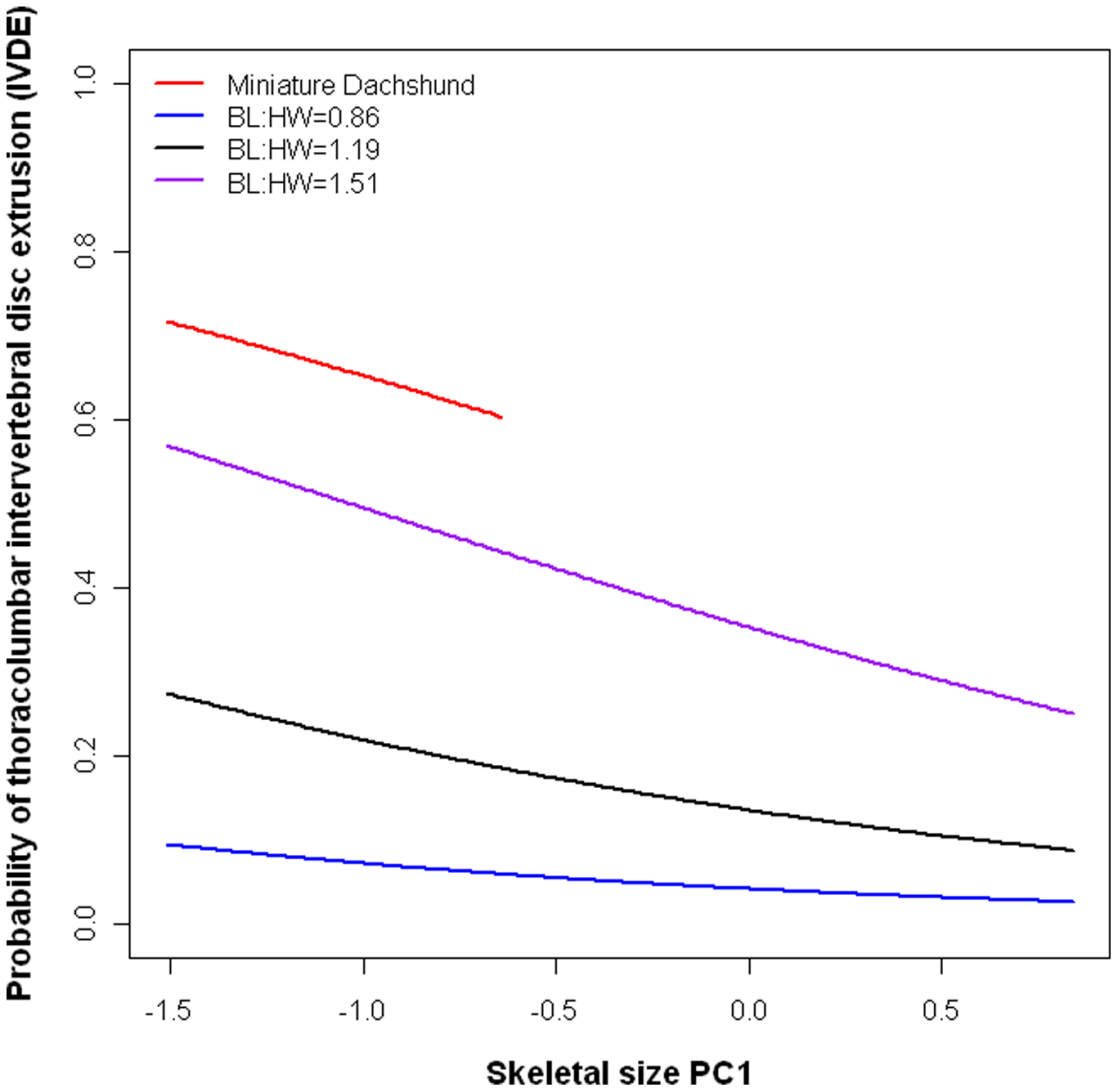
Probability of being affected by thoracolumbar intervertebral disc extrusion across the PC1 (size) spectrum. Miniaturisation is demonstrated as a risk factor for intervertebral disc extrusion. As PC1 increases (and therefore skeletal size increases), the probability of being affected decreases in all breeds. Age = 5 years and BCS = 5 for all dogs, with BL:HW = 1.5 (breed mean) for Miniature Dachshunds (red), and BL:HW = 0.86, 1.19 and 1.51 for the three hypothetical cross breed comparisons, to demonstrate this relationship in relatively short (blue), relatively long (black), and extremely relatively long (purple) dogs. Breed estimates for the Miniature Dachshunds are limited to values of PC1 recorded in the study population, for biological plausibility.

Dogs with smaller skeletal sizes, as summarised using PC1, were more at risk of thoracolumbar IVDE (P = 0.002; Affected mean±SE = −0.67±0.01; Unaffected = 0.13±0.001). Example estimates are modelled for the Miniature Dachshund and three hypothetical cross breed morphologies; of relatively short, relatively long, and extreme relatively long shapes to demonstrate the differing effect of increasing size on disease risk (Figure 3). Of the 15 longest breeds (Table 3), the majority were small breeds, with correspondingly low PC1 values, with the exception of the Basset Hound.

## Discussion

This study provides evidence that relatively longer backs, miniaturisation and obesity are associated with a higher risk of thoracolumbar IVDE in a variety of breeds. It has enabled the first quantitative estimates associating conformation with the risk of disc extrusions, as requested by the Council of Europe (1995), where it was suggested that “*Maximum values for the proportion between length and height of short-legged dogs (e.g. Basset Hound, Dachshund) to avoid disorders of the vertebral column”* should be set [30]. This study has demonstrated the association across a variety of breeds independent of breed-genetic factors, which were accounted for through the random effect of breed in the mixed model analysis. As such, general risk estimates have been modelled, that can be used to guide breeding decisions, to consider the impact of morphology upon the subsequent risk of IVDE of potential offspring. Specific estimates have also been modelled for the Miniature Dachshund breeds due to their high-risk morphology and high representation in this study. Although several high BL:HW ratio breeds (e.g. Standard Dachshund varieties, Pembroke Welsh Corgi) were relatively rare in this study population (and indeed they are relatively rare as breeds generally), they should still be considered high-risk due to their morphology. It is of interest that several more moderately shaped dogs were also affected by IVDE, particularly Jack Russell Terriers (mean BL:HW: 0.99) and Cocker Spaniels (mean BL:HW: 1.04) (Table 3). This may be due to additional non-morphological effects predisposing them to IVDE, for example other genetic factors, or other pathophysiological changes occurring in their discs predisposing them to extrusion.

The estimates here are based on a referral population of companion dogs, and the prevalence of IVDE in the general population is likely to be lower. For example, the overall prevalence of intervertebral disc disease in a UK population survey was 6.8% [23]. That overall prevalence included young dogs outside of the ‘high-risk’ age range of 3–7 years, and when categorised by age group, prevalence in dogs over 10 was higher and more aligned with the existing literature. For example, 38.3% of >10 year old Standard Smooth Haired Dachshunds were affected, and 26.1% Miniature Smooth Haired [23]. There may also be differences between the show dog and companion dog population, or between dogs in different geographical regions that alter risk, which may explain the variability in prevalence estimates. For example, 32% of Miniature Long Haired Dachshunds were affected in this study and 65% of Miniature Smooth Haired Dachshunds; whereas in a US-based study the incidence was ∼19% in Dachshunds generally, but there was a much higher incidence in some Dachshund families where 62% of individuals were affected [19].

It is possible that the Dachshund population in the South East of England is more affected than in previously studied geographical regions; however, the current data were derived from a referral population, and as such it is possible that the absolute prevalences and risk estimates here are inflated due to the inherent biases of such populations. On the other hand, not all dogs in the hospital population underwent advanced diagnostic imaging of the vertebral column, so dogs classed as ‘unaffected’ may actually have been subclinical IVDE cases, or may have had disc degeneration that would lead to IVDE in the future - this could have led to underestimates in the prevalence estimates stated above. Nevertheless, the relative risk relationships, i.e. the shapes of the modelled curves, are likely to mirror those in the general canine population. More refined estimates of the true prevalence would enable adjustment of the model to further improve recommended limits.

Small body size was additionally found to be a risk factor for IVDE, and was independent of breed. It may represent the degree of dwarfism as an additional factor, or may reflect differing biomechanical strains between smaller and larger dogs that alter the level of risk. As dogs become smaller, the relative size of the human environment they live in (e.g. stairs, furniture etc) becomes relatively larger, which may lead to discs being subjected to increased forces, as they jump or fall, for example. They are more likely to be lifted and carried than larger dogs, potentially directly or indirectly causing additional strain of the vertebral column. The marked growth in registrations of small ‘handbag’ dog breeds in recent years [35] is of additional concern in light of small body size being associated with increased risk. Potential purchasers of these breeds should be made aware of the health risks associated with dogs at this extreme end of the size spectrum, and breeding for these extremes should be avoided where possible. In dogs with high BL:HW values, extremely small skeletal size should be avoided so as not to increase risk further. The high BL:HW values (mean BL:HW 1.5) combined with low PC1 values (mean PC1: −1.18) of Miniature Dachshunds may explain their particular predisposition to IVDE. The Jack Russell Terrier, the fourth most affected breed in this study, has a moderate and proportioned morphology (mean BL:HW: 0.99); however, is another miniaturised breed with a low PC1 (mean PC1: −0.96). In comparison, the relatively high BL:HW values of the Basset Hound (mean BL:HW: 1.4) combined with relatively high PC1 values (mean PC1∶0.84) may demonstrate a protective effect of being a larger chondrodystrophic dog.

Overweight dogs were also at an increased risk of IVDE. This has been previously based on anecdote, with weight loss commonly advised by vets as a preventative measure for at risk breeds. Although this effect was not as strong as relative back length (Table 4), excessive weight may be an exacerbating factor, causing additional loading upon calcified intervertebral discs, increasing their risk of extrusion. The effect of increased BCS was demonstrated in the Miniature Dachshund in comparison to cross breeds of a more moderate morphology (Figure 2), highlighting the risk of excessive weight in this breed, and the need to keep dogs at a BCS no higher than 5. The model suggests a decrease in risk as BCS decreases across the whole spectrum of body conditions, but dogs of low BCS were rare in the study population, so the model may not represent them accurately, and low BCS (<4) can be associated with other health problems, and thus should be avoided. A healthy, lean BCS of 4–5 is therefore recommended.

The results of this study indicate that moderating the morphology of extreme chondrodystrophic breeds would reduce their risk of IVDE. This could be achieved through breeding towards more moderate, lower-risk morphologies to bring about reduced prevalences of IVDE over time. At least three approaches could be used towards this aim: (1) only those dogs with more moderate morphologies could be selected for breeding, (2) screening could be used to help select only those more extreme dogs that have no sign of associated disc pathology [15], [36], [37] or genetic disposition [38] for breeding (if such tests are developed), and/or (3) for breeds lacking more moderate morphological variation judicious out-crossing may be considered.

To encourage conformational change, amendments to breed standards are recommended to ensure that breeders working towards these lower-risk morphologies are rewarded in the show-ring. If standards were to be changed, quantitative emphasis on proportions of back length to leg length are required. It has been stated by the Kennel Club that “*The Dachshund is a short-legged dog, not a long-backed one. Excessive length can lead to problems with back disease*” [39]. Although the emphasis here is on the low height of the dog, if back length is kept constant while leg length is shortened, then relative back length will be longer, and proportions altered to become higher risk. In breeds with extreme rectangular shapes that are dictated by actual proportions e.g. the Dandie Dinmont Terrier, where “*heights at the withers should be half the length of the body*” [40], alterations should be made to standards to dictate a more moderate shape. Similarly, in the Fédération Cynologique Internationale (FCI) Dachshund breed standard it is stated that “*the body length should be in harmonious relation to height at withers, about 1 to 1,7–1,8″* [41], and in the UK Kennel Club standard, “*height at the withers should be half the length of the body, measures from breastbone to rear of thigh*” [16]. All these standards suggest extreme long and low morphologies, but they refer to the length of the body rather than the back specifically, so they are not directly comparable to the measurements in this study; focus in all standards should be on back length (withers to sacrum) over body length, because in breeds with prominent sternums (breast bones) body length may be disproportionately influenced by this feature.

Information in this study will be of value to breeders and show-ring judges in deciding what morphologies should be bred for and rewarded. The influence of dog-showing as a tool for change should not be underestimated, and the training and education of judges to ensure that they reward only dogs with healthy morphologies in the show-ring is an area to focus upon. Indeed, it has previously been noted that judges in the show-ring are actively rewarding the long rectangular appearance that is typical of many breeds predisposed both to IVDE, as demonstrated here, and to hip dysplasia [42]. Education of judges has already begun in Sweden, with the Swedish Kennel Club producing breed-specific instructions that highlight morphometric risk factors to judges in at-risk breeds [43]. For example, the Cardigan Welsh Corgi, whose FCI breed standard dictates it should be “*Long in proportion to height*”, has “*exaggeration of body length and excessive shortness of legs*” highlighted as an area of risk in this breed [43]. This is not mentioned for other ‘high-risk’ breeds, such as the Dachshund, Basset Hound and Pekingese, but is equally applicable. The information in this study should also be accessible to existing owners of high-risk breeds; to ensure that they are aware of any environmental or husbandry issues that will be necessary to reduce the risk of IVDE occurring in their dogs, and to new puppy buyers; so they can make informed decisions about selecting breeds.

### Conclusion

This study supports the notion promoted in recent years that exaggerated morphologies are associated with higher risks of associated inherited disorders, and as such breeders should select away from such extreme conformations, towards moderate, safer shapes. Specifically, dogs should not be bred for extremely long backs and extremely small size, if the associated risk of IVDE is to be prevented. Unless prevalence of IVDE risk can be reduced in the most morphologically extreme breeds via other routes, substantial changes may be required in some breed standards to move towards lower risk morphologies.

## References

[1] Bray JP, Burbidge HM (1998) The canine intervertebral disk - Part two: Degenerative changes - Nonchondrodystrophoid versus chondrodystrophoid disks. J Am Anim Hosp Assoc 34: 135–144. 9507426 10.5326/15473317-34-2-135

[2] Levine JM, Budke CM, Levine GJ, Kerwin SC, Hettlich BF, et al (2008) Owner-perceived, weighted quality-of-life assessments in dogs with spinal cord injuries. J Am Vet Med Assoc 233: 931–935. 18795855 10.2460/javma.233.6.931

[3] Freeman PM, Holmes M, Blamires H, Jeffery N, Granger N (2012) Impact on owners of home-based management of dogs with severe chronic spinal cord injury. 25^th^ Symposium of the ESVN-ECVN. Ghent, Belgium.

[4] Bauer M, Glickman N, Glickman L, Toombs J, Golden S, et al (1992) Follow-up study of owner attitudes toward home care of paraplegic dogs. J Am Vet Med Assoc 200: 1809–1816. 1639682

[5] Bergknut N, Egenvall A, Hagman R, Gustås P, Hazewinkel HAW, et al (2012) Incidence of intervertebral disk degeneration–related diseases and associated mortality rates in dogs. J Am Vet Med Assoc 240: 1300–1309. 22607596 10.2460/javma.240.11.1300

[6] Braund KG, Ghosh P, Taylor TKF, Larsen LH (1975) Morphological Studies of Canine Intervertebral-Disk - Assignment of Beagle to Achondroplastic Classification. Res Vet Sci 19: 167–172. 1166121

[7] Gage ED (1975) Incidence of clinical disc disease in the dog. J Am Anim Hosp Assoc 11: 135–138.

[8] Cappello R, Bird JLE, Pfeiffer D, Bayliss MT, Dudhia J (2006) Notochordal cell produce and assemble extracellular matrix in a distinct manner, which may be responsible for the maintenance of healthy nucleus pulposus. Spine 31: 873–882. 16622374 10.1097/01.brs.0000209302.00820.fd

[9] Braund KG (1993) Intervertebral Disc Disease. In: Bojrab M, Smeak DD, Bloomberg MS, editors. Disease Mechanisms in Small Animal Surgery. 2nd Ed. Philadelphia: Lea and Febiger. 960–970.

[10] Hansen HJ (1952) A pathological-anatomical study on disc degeneration in the dog. Acta Orthop Scand Suppl 11: 1–120. 10.3109/ort.1952.23.suppl-11.01 14923291

[11] Bergknut N, Rutges JP, Kranenburg HJ, Smolders LA, Hagman R, et al (2012) The dog as an animal model for intervertebral disc degeneration? Spine 37: 351–358. 21544011 10.1097/BRS.0b013e31821e5665

[12] Bergknut N, Meij BP, Hagman R, de Nies K, Rutges JP, et al (2013) Intervertebral disc disease in dogs - Part 1: A new histological grading scheme for classification of intervertebral disc degeneration in dogs. Vet J 195: 156–163. 22789628 10.1016/j.tvjl.2012.05.027

[13] Kranenburg HC, Grinwis GCM, Bergknut N, Gahrmann N, Voorhout G, et al (2012) Intervertebral disc disease in dogs: Part 2: Comparison of clinical, magnetic resonance imaging, and histological findings in 74 surgically treated dogs. Vet J 195: 164–171. 22795604 10.1016/j.tvjl.2012.06.001

[14] Smolders L, Bergknut N, Grinwis G, Hagman R, Lagerstedt A, et al (2013) Intervertebral disc degeneration in the dog. Part 2: Chondrodystrophic and non-chondrodystrophic breeds. Vet J 195: 292–299. 23154070 10.1016/j.tvjl.2012.10.011

[15] Jensen VF, Beck S, Christensen KA, Arnbjerg J (2008) Quantification of the association between intervertebral disk calcification and disk herniation in Dachshunds. J Am Vet Med Assoc 233: 1090–1095. 18828719 10.2460/javma.233.7.1090

[16] The Kennel Club (2009) Dachshund (Long-Haired) Breed Standard. Available: http://www.the-kennel-club.org.uk/services/public/breed/standard.aspx?id=1009. Accessed 1 November 2012.

[17] The Kennel Club (2010) Basset Hound Breed Standard. Available: http://www.the-kennel-club.org.uk/services/public/breed/standard.aspx?id=1003. Accessed 1 November 2012.

[18] Parker HG, VonHoldt BM, Quignon P, Margulies EH, Shao S, et al (2009) An Expressed Fgf4 Retrogene Is Associated with Breed-Defining Chondrodysplasia in Domestic Dogs. Science 325: 995–998. 19608863 10.1126/science.1173275 PMC2748762

[19] Ball MU, Mcguire JA, Swaim SF, Hoerlein BF (1982) Patterns of Occurrence of Disk Disease among Registered Dachshunds. J Am Vet Med Assoc 180: 519–522. 7061336

[20] Jensen VF, Ersboll AK (2000) Mechanical factors affecting the occurrence of intervertebral disc calcification in the dachshund–a population study. J Vet Med A Physiol Pathol Clin Med 47: 283–296. 10932525 10.1046/j.1439-0442.2000.00296.x

[21] Verheijen J, Bouw J (1982) Canine intervertebral disc disease: a review of etiologic and predisposing factors. Vet Q 4: 125–134. 6755879 10.1080/01652176.1982.9693852

[22] Priester WA (1976) Canine intervertebral disc disease – Occurrence by age, breed, and sex among 8,117 cases. Theriogenology 6: 293–303.

[23] Dachshund Breed Council (2012) Dachs-Life 2012: Report on the Dachshund Breed Council’s Health Survey. Available: http://dachshundbreedcouncil.files.wordpress.com/2012/05/dachs-life2012report1.pdf. Accessed 1 November 2012.

[24] Brisson BA, Moffatt SL, Swayne SL, Parent JM (2004) Recurrence of thoracolumbar intervertebral disk extrusion in chondrodystrophic dogs after surgical decompression with or without prophylactic fenestration: 265 cases (1995–1999). J Am Vet Med Assoc 224: 1808–1814. 15198267 10.2460/javma.2004.224.1808

[25] Mayhew PD, McLear RC, Ziemer LS, Culp WT, Russell KN, et al (2004) Risk factors for recurrence of clinical signs associated with thoracolumbar intervertebral disk herniation in dogs: 229 cases (1994–2000). J Am Vet Med Assoc 225: 1231–1236. 15521446 10.2460/javma.2004.225.1231

[26] Olby N, Levine J, Harris T, Munana K, Skeen T, et al (2003) Long-term functional outcome of dogs with severe injuries of the thoracolumbar spinal cord: 87 cases (1996–2001). J Am Vet Med Assoc 222: 762–769. 12675299 10.2460/javma.2003.222.762

[27] Scott HW, McKee WM (1999) Laminectomy for 34 dogs with thoracolumbar intervertebral disc disease and loss of deep pain perception. J Small Anim Pract 40: 417–422. 10516947 10.1111/j.1748-5827.1999.tb03114.x

[28] Levine JM, Levine GJ, Kerwin SC, Hettlich BF, Fosgate GT (2006) Association between various physical factors and acute thoracolumbar intervertebral disk extrusion or protrusion in Dachshunds. J Am Vet Med Assoc 229: 370–375. 16881827 10.2460/javma.229.3.370

[29] Sharp NJH, Wheeler SJ (2005) Thoracolumbar disc disease. In: Sharp NJH, Wheeler SJ, editors. Small Animal Spinal Disorders: Diagnosis and Surgery. Philadelphia: Elsevier Mosby. 106–210.

[30] Council of Europe (1995) Resolution on the breeding of pet animals, Multilateral Consultation of parties to the European Convention for the protection of pet animals (ETS 123), March 1995 in Strasbourg, Document CONS 125(95)29, Council of Europe, F 67075 Strasbourg-Cedex.

[31] Sutter NB, Mosher DS, Gray MM, Ostrander EA (2008) Morphometrics within dog breeds are highly reproducible and dispute Rensch’s rule. Mamm Genome 19: 713–723. 19020935 10.1007/s00335-008-9153-6 PMC2748280

[32] Purina (2012) Assessing your dog’s body condition. Available: http://www.purina.co.uk/content/your-dog/helping-to-keep-your-dog-healthy/managing-your-dog%27s-weight/assessing-your-dog%27s-body-condition. Accessed 1 November 2012.

[33] Parker H, Kim L, Sutter N, Carlson S, Lorentzen T, et al (2004) Genetic structure of the purebred domestic dog. Science 304: 1160–1164. 15155949 10.1126/science.1097406

[34] The Kennel Club (2006) Kennel Club Breed Standards. Available: http://www.thekennelclub.org.uk/item/210. Accessed 1 November 2012.

[35] The Kennel Club (2011) Celebrity Handbag Dog Registrations Soar As Pug Makes Top Ten For The First Time. Available: http://www.thekennelclub.org.uk/item/3535/23/5/3. Accessed 1 November 2012.

[36] Havranek-Balzaretti B (1980) Beitrag zur Aetiologie der Dackellähme und Vorschlag zur züchterischen Selektion. (Etiology of the intervertebral disc disease in dachshunds and suggestions for breeding). Zürich: Veterinär-Chirurgischen Klinik und Institut für Veterinärpatologie. Dissertation, Universität Zürich.

[37] Rohdin C, Jeserevic J, Viitmaa R, Cizinauskas S (2010) Prevalence of radiographic detectable intervertebral disc calcifications in Dachshunds surgically treated for disc extrusion. Acta Vet Scand 52: 24. 20398282 10.1186/1751-0147-52-24 PMC2873269

[38] Mogensen MS, Karlskov-Mortensen P, Proschowsky HF, Lingaas F, Lappalainen A, et al (2011) Genome-wide association study in Dachshund: identification of a major locus affecting intervertebral disc calcification. J Hered 102 Suppl 1S81–86. 21846751 10.1093/jhered/esr021

[39] The Kennel Club (2012) Breed Information Centre: Dachshund (Long-Haired) Description. Available: http://www.the-kennel-club.org.uk/services/public/breed/display.aspx?id=1009. Accessed 1 November 2012.

[40] The Kennel Club (2000) Dandie Dinmont Terrier Breed Standard. Available: http://www.the-kennel-club.org.uk/services/public/breed/standard.aspx?id=3066. Accessed 1 November 2012.

[41] Fédération Cynologique Internationale (2001) FCI-Standard N° 148/13. 07. 2001/GB. Available: www.fci.be/uploaded_files/148GB99_en.doc. Accessed 1 November 2012.

[42] Roberts T, McGreevy PD (2010) Selection for breed-specific long-bodied phenotypes is associated with increased expression of canine hip dysplasia. Vet J 183: 266–272. 19959383 10.1016/j.tvjl.2009.11.005

[43] Svenska Kennelklubben (2010) Special Breed Specific Instructions (BSI) regarding exaggerations in pedigree dogs. Available: http://www.skk.se/Global/Dokument/Utstallning/special-breed-specific-instructions-A8.pdf. Accessed 1 November 2012.

[44] Roach WJ, Thomas M, Weh JM, Bleedorn J, Wells K (2012) Residual herniated disc material following hemilaminectomy in chondrodystrophic dogs with thoracolumbar intervertebral disc disease. Vet Comp Orthop Traumatol 25: 109–115. 22286190 10.3415/VCOT-11-05-0075

[45] Trębacz P, Sterna J, Trębacz E, Bonecka J (2011) Simultaneous acute intervertebral disk extrusion in two adjacent intervertebral spaces in dogs. Medycyna Weterynaryjna 67: 634–638.

[46] Brisson BA, Holmberg DL, Parent J, Sears WC, Wick SE (2011) Comparison of the effect of single-site and multiple-site disk fenestration on the rate of recurrence of thoracolumbar intervertebral disk herniation in dogs. J Am Vet Med Assoc 238: 1593–1600. 21671814 10.2460/javma.238.12.1593

[47] Mateo I, Lorenzo V, Foradada L, Munoz A (2011) Clinical, pathologic, and magnetic resonance imaging characteristics of canine disc extrusion accompanied by epidural hemorrhage or inflammation. Vet Radiol Ultrasound 52: 17–24. 21322383

[48] Forterre F, Dickomeit M, Senn D, Gorgas D, Spreng D (2011) Microfenestration using the CUSA Excel ultrasonic aspiration system in chondrodystrophic dogs with thoracolumbar disk extrusion: a descriptive cadaveric and clinical study. Vet Surg 40: 34–39. 21175695 10.1111/j.1532-950X.2010.00780.x

[49] Johnson JA, da Costa RC, Allen MJ (2010) Micromorphometry and cellular characteristics of the canine cervical intervertebral discs. J Vet Intern Med 24: 1343–1349. 20946372 10.1111/j.1939-1676.2010.0613.x

[50] Brisson BA (2010) Intervertebral Disc Disease in Dogs. Vet Clin North Am Small Anim Pract 40: 829–858. 20732594 10.1016/j.cvsm.2010.06.001

[51] Lim C, Kweon OK, Choi MC, Choi J, Yoon J (2010) Computed tomographic characteristics of acute thoracolumbar intervertebral disc disease in dogs. J Vet Sci 11: 73–79. 20195068 10.4142/jvs.2010.11.1.73 PMC2833433

[52] Forterre F, Lang J (2010) New aspects in the treatment of disc herniation in the dog. Schweizer Archiv Fur Tierheilkunde 152: 109–113. 20235010 10.1024/0036-7281/a000027

[53] King JB, Jones JC, Rossmeisl JH, Harper TA, Lanz OI, et al (2009) Effect of multi-planar CT image reformatting on surgeon diagnostic performance for localizing thoracolumbar disc extrusions in dogs. J Vet Sci 10: 225–232. 19687623 10.4142/jvs.2009.10.3.225 PMC2801132

[54] Hecht S, Thomas WB, Marioni-Henry K, Echandi RL, Matthews AR, et al (2009) Myelography vs. computed tomography in the evaluation of acute thoracolumbar intervertebral disk extrusion in chondrodystrophic dogs. Vet Radiol Ultrasound 50: 353–359. 19697598 10.1111/j.1740-8261.2009.01549.x

[55] Malik Y, Spreng D, Konar M, Doherr MG, Jaggy A, et al (2009) Laser-Doppler measurements of spinal cord blood flow changes during hemilaminectomy in chondrodystrophic dogs with disk extrusion. Vet Surg 38: 457–462. 19538666 10.1111/j.1532-950X.2009.00529.x

[56] Erwin WM (2008) The Notochord, Notochordal cell and CTGF/CCN-2: ongoing activity from development through maturation. J Cell Commun Signal 2: 59–65. 19003520 10.1007/s12079-008-0031-5 PMC2648046

[57] Itoh H, Hara Y, Yoshimi N, Harada Y, Nezu Y, et al (2008) A retrospective study of intervertebral disc herniation in dogs in Japan: 297 cases. J Vet Med Sci 70: 701–706. 18685242 10.1292/jvms.70.701

[58] Probst A, Modler F, Kunzel W, Mlynarik V, Trattnig S (2008) Demonstration of the articular cartilage of the canine ulnar trochlear notch using high-field magnetic resonance imaging. Vet J 177: 63–70. 17513147 10.1016/j.tvjl.2007.03.018

[59] Forterre F, Konar M, Spreng D, Jaggy A, Lang J (2008) Influence of intervertebral disc fenestration at the herniation site in association with hemilaminectomy on recurrence in chondrodystrophic dogs with thoracolumbar disc disease: a prospective MRI study. Vet Surg 37: 399–405. 18564265 10.1111/j.1532-950X.2008.00394.x

[60] Itoh H, Asou Y, Hara Y, Haro H, Shinomiya K, et al (2008) Enhanced type X collagen expression in the extruded nucleus pulposus of the chondrodystrophoid dog. J Am Vet Med Assoc 70: 37–42. 10.1292/jvms.70.37 18250570

[61] Sterna J, Burzykowski T (2008) Assessment of the usefulness of the fenestration method in cases of disc extrusion in the cervical and thoraco-lumbar spine in chondrodystrophic dogs. Pol J Vet Sci 11: 55–62. 18540209

[62] Sterna J (2007) The assessment of ventral cervical decompression in the treatment of prolapse of the nucleus pulposus in dogs. Pol J Vet Sci 10: 89–95. 17882932

[63] Stigen O, Ottesen N, Jaderlund KH (2010) Early recurrence of thoracolumbar intervertebral disc extrusion after surgical decompression: a report of three cases. Acta Vet Scand 52: 10. 20137085 10.1186/1751-0147-52-10 PMC2829575

[64] Sakai D, Nakai T, Mochida J, Alini M, Grad S (2009) Differential phenotype of intervertebral disc cells: microarray and immunohistochemical analysis of canine nucleus pulposus and anulus fibrosus. Spine (Phila Pa 1976) 34: 1448–1456. 19525835 10.1097/BRS.0b013e3181a55705

[65] Ryan TM, Platt SR, Llabres-Diaz FJ, McConnell JF, Adams VJ (2008) Detection of spinal cord compression in dogs with cervical intervertebral disc disease by magnetic resonance imaging. Vet Rec 163: 11–15. 18603629 10.1136/vr.163.1.11

[66] Degórska B, Sterna J, Sapierzynski R, Siedlicki M (2009) What is your diagnosis? Cervical pain and paresis of left thoracic limb in a chondrodystrophic dog. Vet Comp Orthop Traumatol 22: 294. 19718847

